# Consent in radiology practice

**Published:** 2009-02

## Consent

There are many instances where consent is necessary in radiology practice

1. Before administering intravenous contrast
2. Before interventional procedures of all types
3. Before administering anesthesia

In all such situations, it is necessary to follow proper procedure regarding consent.

1. The consent form itself must be comprehensive and cover all issues of importance, including major and common complications, based on an explanation given to the patient and his/her relatives/friends, in their own language
2. The signature or thumb impression of the patient must be taken
3. A relative or accompanying person must endorse this consent at the same time
4. No changes must be made to the consent form thereafter

As the two cases reproduced here show, an improperly filled consent form can lead to significant problems, if there is a medico-legal issue in the future. Conversely, a properly filled consent form can protect the doctor from future problems as well

## Consent form – One person, one sitting, one pen

Dr. AKG v/s Hospital & Anr.

Reproduced with permission from Medical Law Cases for Doctors, Vol 1, Issue 10, October 2008

## Facts of the case

The patient, an 85-year-old man, was operated for prostatic enlargement. The operating doctors (OP's) first adopted the Trans Urethral Resection of Prostate (TURP – ‘pin hole’) method, but subsequently switched over to a trans-vesical route (open surgical method). The patient was moved to the recovery room and thereafter to his ward. Complications arose a few hours later. There was some delay in the arrangement and transfusion of blood. The condition of the patient started deteriorating. He was given oxygen but expired the same evening.

## Patient's allegation/s of medical negligence

- It was alleged that the patient had signed a blank consent form on which TURP had been written later. Moreover, TURP was written in the wrong column of the consent form.
- It was alleged that the Urologist (OP 2) had clearly indicated that the patient would be operated upon by the trans-vesical method. But during surgery, he adopted the TURP method. This was a big mistake, since the prostate was diagnosed to be large, of about 60 g in size, which could be removed only by open prostatectomy.
- It was alleged that there was excessive bleeding, which could not be controlled as preoperative tests of clotting time/bleeding time (CT/BT), were not done.
- It was alleged that adequate management for sufficient blood for transfusion had not been made. Moreover, there was a delay of three hours due to the attitude of the doctor on emergency duty who raised flimsy objections.

## Doctor's defense

- It was stated in defense that the initial choices of TURP and subsequent change to the trans-vesical method were both professionally a sound decision. The TURP method involves minimum incision and disruption. On examining the patient, the prostate gland appeared to be of a size, which could be removed by the TURP method. But prior examination of the patient does not always give an accurate estimate of the size of the prostate gland. Medical literature was produced before the court, which stated that the size of the gland that can be removed by the TURP method depends on the training, experience, temperament, skill, and competence of the surgeon and no hard and fast rule can be laid down.
- Medical literature was produced which stated that in the absence of clinical evidence of a hemostatic disorder there is only a 0.008% probability that any given patient will have an interoperative clotting disorder. Further, the patient and even his son who was a doctor, had given no history of any such disorder.
- It was stated that the postoperative advice of the anesthetist was to keep watch on vital parameters. Blood was to be transfused only if it was indicated by the hemoglobin test report. As the blood pressure was normal and steady, blood transfusion did not seem to be urgent. It was transfused only because the patient's doctor son, on his own, desired blood transfusion as he found the tongue of the patient to be white, without waiting for the hemoglobin test report.

## Findings of the court

- The court perused the case papers of the patient other than the consent form, and found that ‘TURP/Open’ was mentioned clearly, everywhere. Hence the court disbelieved the allegations that the word ‘TURP’ was inserted by OP2 on a blank consent form. Moreover, the court also drew adverse inference against the patient as the word ‘TURP’ was written by the same hand that had filled the consent form.
- The court upheld the decision of the operating doctors (OP's) to subsequently change to the trans-vesical method as it was a professional choice available to an operating surgeon. The court further held that even the best option may turn out to be an error of judgment, which cannot be construed as negligence.
- The court relied on medical literature that clearly stated that hemostatic disorders are very rare. As the patient had not indicated any bleeding disorder at the time of examination, the court held that the omission to conduct the CT/BT test was not negligence.
- The court observed that merely because the patient's son happened to be a doctor, his authentication could not substitute the cross-matching test and the compatibility certification given by a qualified blood bank technician of the hospital (OP). (It seems the patient's son sought a blood transfusion without blood been cross-checked by the hospital's blood bank.)
- Hence the hospital and the doctors (OP) were held not negligent.

## Suggested Precautions

1. Consent form must be filled by one doctor/nurse, in one sitting, if possible without changing the pen though counseling the patient may take more than one sitting
2. In the consent form, carefully make entries at the appropriate spaces. Entries made at the wrong place raise suspicion.
3. In case alternative procedures have already been contemplated, it is advisable to clearly specify each of these procedures in the consent form.
4. Specific consent for each and every type of anesthesia that is anticipated must be taken.
5. Hospitals and Nursing Homes must politely refuse anyone, even another qualified doctor, from interfering. (In this case, the patient's son was a qualified doctor. It seems his insistence to transfuse blood without cross-matching was refused by the hospital staff and this action was upheld by the court.)
6. Only a qualified blood bank technician must do cross-matching and certify compatibility of blood.

